# Clinical Applications and Evidence Landscape of Thymosin Alpha 1 as an Immunomodulatory Adjunct in Cancer Care: A Scoping Review

**DOI:** 10.3390/ph19091492

**Published:** 2026-09-19

**Authors:** Soo-Dam Kim, Kyu Ri Kim, Dong-Hyeon Kim, Taekyung Yeo, Mi-Jeong Choi, So-Jung Park, Hwa-Seung Yoo

**Affiliations:** 1 Department of Integrative Medicine, JABA Graduate School, Wonkwang University, Iksan 54538, Republic of Korea; hidden425@wku.ac.kr; 2 East West Cancer Center, Daejeon Korean Medicine Hospital, Daejeon University, Daejeon 34520, Republic of Korea; startown1110@naver.com; 3 Korean Medicine Hospital, Pusan National University, Yangsan 50612, Republic of Korea; dongxian92@gmail.com; 4 Oculi Korean Medicine Hospital, Seoul 05655, Republic of Korea; oculi2022@naver.com; 5 Biomedical Biotechnology Research Institute Co., Ltd., Goyang-si 10326, Republic of Korea; choimijeong5@gmail.com; 6 Department of Korean Internal Medicine, School of Korean Medicine, Pusan National University, Yangsan 50612, Republic of Korea

**Keywords:** thymosin alpha 1, thymalfasin, cancer, immunomodulation, adjunctive therapy, clinical evidence

## Abstract

**Background**: Thymosin alpha 1 (Tα1, thymalfasin) is a thymus-derived immunomodulatory peptide investigated as an adjunctive therapy in cancer care. Although studied across malignancies, its evidence landscape has not been systematically mapped. This scoping review characterized treatment contexts, intervention patterns, outcome domains, and safety reporting of clinical studies evaluating Tα1 in patients with cancer. **Methods**: PubMed, Embase, and CENTRAL were searched for clinical studies of Tα1 in patients with cancer published from January 2016 to March 2026. Eligible studies included randomized controlled trials, non-randomized studies, observational studies, and case-based reports. Data on study characteristics, cancer type, intervention features, comparators, outcomes, and adverse-event reporting were charted and synthesized descriptively. **Results**: Twenty-six clinical publications were included. Most were conducted in China, and retrospective observational studies and case reports predominated. Hepatobiliary, thoracic, and gastrointestinal cancers were the most frequently studied clusters. Classical Tα1 was administered mainly by subcutaneous injection, most commonly at 1.6 mg, and used primarily as an adjunct to chemotherapy, radiotherapy, chemoradiotherapy, targeted therapy, immune checkpoint inhibitors, or postoperative and supportive care. Outcomes included tumor response, survival, recurrence, immune and inflammatory markers, treatment-related toxicity, functional status, and quality of life. Several studies reported favorable clinical, immune-related, or treatment-tolerance signals, but heterogeneity limited comparability. Safety reporting was inconsistent. One case described severe multisystem immune-related toxicity during combination treatment without establishing Tα1-specific causality. **Conclusions**: Tα1 has been investigated mainly as an adjunctive immunomodulatory strategy in cancer care. Prospective controlled studies with standardized dosing, defined treatment contexts, consistent outcomes, and systematic harms reporting are needed.

## 1. Introduction

Cancer remains a major global public health challenge, with its burden continuing to increase alongside population growth and aging. Recent estimates indicate that 20.6 million new cancer cases and 9.8 million cancer deaths occurred worldwide in 2024, with annual cancer incidence projected to increase to 34.4 million cases by 2050 [1]. The oncological treatment landscape has evolved from conventional cytotoxic chemotherapy toward molecularly targeted agents and immunotherapies [2]. In particular, immune checkpoint inhibitors (ICIs) have changed cancer treatment by restoring antitumor immune responses and producing durable clinical benefit in selected patients [2]. However, many patients do not respond adequately or eventually develop resistance, and immune-related adverse events can compromise treatment continuity [3,4].

Thymosin alpha 1 (Tα1, thymalfasin) is a synthetic 28-amino acid peptide corresponding to a naturally occurring thymic peptide [5,6]. Tα1 has been reported to regulate both innate and adaptive immunity through effects on T-cell maturation and differentiation, dendritic-cell activation, natural killer-cell function, cytokine regulation, and immune homeostasis [5,7,8]. Because of these immunomodulatory properties, Tα1 has been used or investigated in chronic viral infections, immune dysfunction, severe infectious diseases, and cancer-related immune suppression [5,9]. In oncology, Tα1 is generally regarded not as a direct cytotoxic anticancer agent, but as an immune-restorative or immune-supportive intervention that may complement conventional and modern anticancer therapies. This immune-supportive profile provides a rationale for investigating Tα1 as an adjunctive strategy to improve host immune competence and treatment tolerance in cancer care [6,7,8].

Mechanistic evidence further supports the potential role of Tα1 in cancer immunomodulation [10]. Experimental studies suggest that Tα1 may influence the tumor microenvironment by modulating immune-cell phenotypes and reducing immunosuppressive signaling [8,10]. In particular, Tα1 has been shown to reverse M2 polarization of tumor-associated macrophages during efferocytosis, thereby promoting a more pro-inflammatory and antitumor immune state [10]. Reviews of Tα1 in cancer therapy have also suggested potential interactions with T-cell function, dendritic-cell activity, cytokine networks, and immune checkpoint-related pathways. These findings indicate that Tα1 may have a biologically plausible role as a synergistic partner in the immuno-oncology era, particularly in treatment settings where immune suppression, lymphopenia, or impaired host response may influence clinical outcomes [5,6,8].

Clinical studies of Tα1 in patients with cancer have accumulated over several decades across different malignancies and treatment settings [5,7,9]. An early randomized study in lung cancer evaluated the immunorestorative properties of synthetic Tα1 in patients with cancer [11]. Subsequent studies investigated Tα1 in hepatocellular carcinoma, including combinations with transcatheter arterial chemoembolization and postoperative treatment strategies [12,13,14,15]. A large randomized study in metastatic melanoma evaluated Tα1, interferon alfa, or both in combination with dacarbazine, showing that Tα1 had already been explored as part of systemic anticancer treatment strategies before the current immunotherapy era [16]. More recent studies have expanded its application to EGFR-mutant non-small cell lung cancer, resected non-small cell lung cancer, locally advanced non-small cell lung cancer, platinum-resistant ovarian cancer, unresectable hepatocellular carcinoma, and advanced metastatic solid tumors [17,18,19,20,21,22].

Despite these expanding clinical applications, the overall evidence landscape of Tα1 in cancer care remains difficult to interpret. Existing studies differ substantially in cancer type, disease stage, treatment context, study design, Tα1 dosage, administration schedule, concomitant therapy, comparator, and outcome measures. Reported outcomes in previous clinical reports and reviews have included tumor response, survival, recurrence, immune-cell profiles, inflammatory markers, treatment-related toxicity, infection, functional status, and quality of life [7,8,9]. Because the existing evidence is heterogeneous and dispersed across diverse malignancies, treatment contexts, and outcome domains, it is necessary to clarify where research activity is concentrated, which clinical settings have been repeatedly evaluated, and which areas remain exploratory. This review aims to map the clinical evidence of Tα1 in cancer care by cancer type, study design, treatment setting, intervention pattern, comparator, outcome domain, and safety reporting, thereby clarifying its current position as an adjunctive immunomodulatory strategy and informing future evidence synthesis and clinical trial design.

## 2. Materials and Methods

### 2.1. Study Design and Framework

This scoping review was designed to systematically map the clinical research landscape of Tα1 in cancer care. The review aimed to summarize study characteristics, cancer types, treatment contexts, intervention patterns, comparators, outcome domains, and safety reporting, thereby identifying areas of repeated investigation and gaps requiring future evidence synthesis or prospective clinical research. The methodological framework originally proposed by Arksey and O’Malley was followed, with additional guidance from the Joanna Briggs Institute approach to strengthen methodological rigor [23,24]. Reporting was guided by the Preferred Reporting Items for Systematic Reviews and Meta-Analyses Extension for Scoping Reviews (PRISMA-ScR) [25]. The protocol was registered with the Open Science Framework on 10 March 2026 (OSF Preregistration; https://doi.org/10.17605/OSF.IO/AM46J, accessed on 18 September 2026). Consistent with scoping review methodology, formal risk-of-bias assessment and certainty-of-evidence appraisal were not conducted, because the purpose of this review was to map the evidence rather than to estimate treatment effects.

### 2.2. Definition of Tα1 Interventions

For this review, classical Tα1 was defined as thymosin alpha 1 or thymalfasin administered as a therapeutic or supportive intervention in patients with cancer. Tα1-containing fusion proteins and mixed thymic-peptide exposures were considered separately because they are not pharmacologically equivalent to classical Tα1. Studies were eligible regardless of formulation, route of administration, dosage, frequency, or treatment duration, provided that Tα1 was used as an intervention or a major component of the treatment regimen. Tα1 could be administered as monotherapy or as part of a combination strategy with anticancer treatment or supportive care. Combination strategies included chemotherapy, radiotherapy, concurrent chemoradiotherapy, targeted therapy, immune checkpoint inhibitors, antiviral therapy, nutritional support, traditional East Asian medicine, hyperthermia, or other integrative supportive interventions. When available, details on formulation, dose, administration schedule, treatment duration, comparator, and concomitant therapies were extracted as reported in the original studies.

### 2.3. Research Questions

This review addressed the following questions: (1) What clinical cancer settings have been investigated for Tα1, and how are the studies distributed by cancer type, country, treatment setting, and study design? (2) What intervention patterns have been used, including route of administration, dose, frequency, duration, concomitant therapies, and comparators? (3) What clinical, immune-related, patient-centered, and safety outcomes have been reported, and which areas appear repeatedly studied or remain exploratory?

### 2.4. Eligibility Criteria

Studies were included if they met the following criteria: (1) involved patients with cancer, regardless of age, sex, cancer type, disease stage, or treatment setting; (2) evaluated Tα1 as an intervention or as a major treatment component; (3) reported clinical outcomes, safety outcomes, or both; and (4) were original clinical studies published in peer-reviewed journals from January 2016 to 10 March 2026. This publication window was selected to characterize recent clinical research trends in Tα1 use in cancer care. Eligible study designs included randomized controlled trials, non-randomized clinical studies, prospective observational studies, retrospective observational studies, case series, and case reports.

Studies were excluded if they met any of the following criteria: (1) non-human experimental studies, including in vitro or animal studies; (2) review articles, meta-analyses, protocols, editorials, commentaries, or other non-original research; (3) conference abstracts or records without accessible full text; (4) studies in which Tα1 was not a primary intervention or major treatment component; or (5) studies not involving patients with cancer. Eligibility was restricted to English-language publications.

### 2.5. Information Sources and Search Strategy

A systematic search was conducted in PubMed, Embase, and the Cochrane Central Register of Controlled Trials (CENTRAL). The database search was performed on 10 March 2026. The search strategy combined cancer-related terms and Tα1-related terms using controlled vocabulary and free-text keywords. Representative cancer-related terms included “cancer,” “carcinoma,” “neoplasm,” “tumor,” “tumour,” and “malignancy.” Representative Tα1-related terms included “Thymosin alpha 1,” “Thymosin alpha-1,” and “Thymalfasin.” Search terms were adapted to the syntax of each database and combined using appropriate Boolean operators. The complete database-specific search strategies are provided in Supplementary Table S1.

### 2.6. Study Selection Process

All records retrieved from the database searches were imported into reference management software, and duplicates were removed before screening. Two reviewers (K.R.K. and S.D.K.) independently screened titles and abstracts according to the predefined eligibility criteria. Full texts of potentially eligible studies were then assessed to determine final inclusion. Any disagreements between reviewers were resolved through discussion, and unresolved discrepancies were adjudicated by a third reviewer (H.S.Y.). The study selection process was summarized using a PRISMA flow diagram, including the numbers of records identified, screened, assessed for eligibility, excluded with reasons, and included in the final review.

### 2.7. Data Extraction and Synthesis

Data extraction was independently performed by two reviewers using a standardized data extraction form developed a priori in Microsoft Excel, Version 2608 (Microsoft Corporation, Redmond, WA, USA). Extracted information included first author, publication year, country, study design, cancer type, organ-system category, disease stage or treatment setting, sample size, participant age, Tα1 formulation, route of administration, dose, frequency, treatment duration, comparator, concomitant therapies, outcome measures, main findings, and adverse-event reporting. Disagreements during data extraction were resolved through discussion or consultation with a third reviewer.

For descriptive synthesis, included studies were grouped by study design, country, cancer type or organ-system category, and treatment context. Classical Tα1, TNF-α–Tα1 fusion proteins, and mixed thymic-peptide exposures with inseparable Tα1-specific outcomes were synthesized separately. Tα1 interventions were summarized according to route of administration, dosage, frequency, treatment duration, treatment role, and concomitant therapy. Treatment role was categorized according to whether Tα1 was used alone, as an adjunct to conventional anticancer therapy, as part of multimodal immunotherapy, or as supportive/integrative care. Outcome measures were grouped into tumor response, survival or recurrence outcomes, immune-cell or inflammatory biomarker outcomes, treatment-related toxicity outcomes, functional or quality-of-life outcomes, and safety outcomes. Frequency analyses were used to summarize the distribution of study designs, cancer types, intervention characteristics, comparator categories, and outcome domains. Narrative summaries were used to identify recurring clinical patterns and research gaps within the mapped evidence. Comparative studies were interpreted according to their design, whereas case reports were used to describe clinical contexts and generate hypotheses rather than establish treatment effects.

## 3. Results

### 3.1. Study Selection

**Figure 1:**
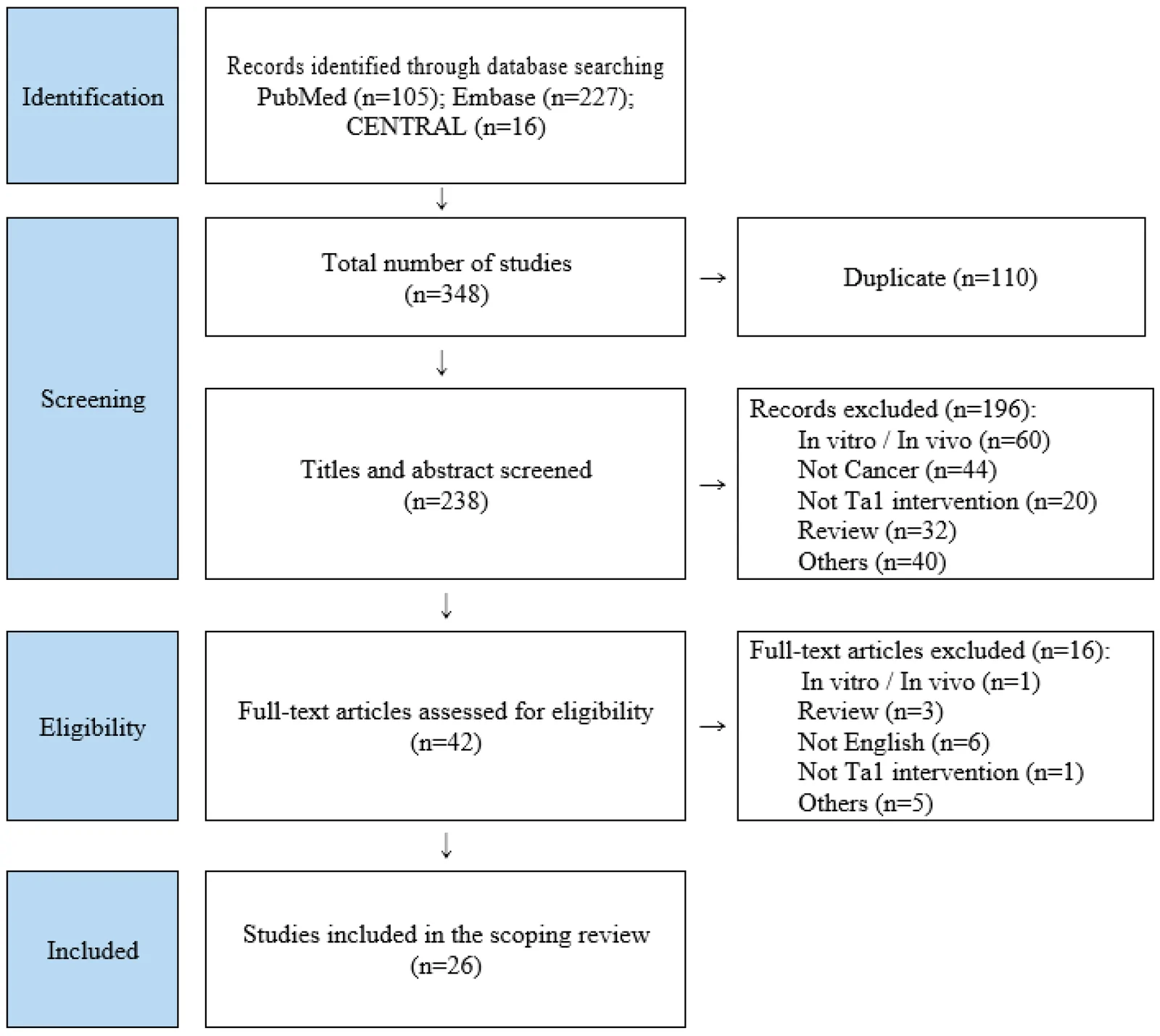
PRISMA-ScR flow diagram of study selection.

The database search identified 348 records, including 105 from PubMed, 227 from Embase, and 16 from CENTRAL. After removing 110 duplicates, 238 records were screened by title and abstract. Of these, 196 records were excluded because they were in vitro or in vivo studies, were not cancer-related, did not evaluate Tα1 as an intervention, were review articles, or met other exclusion criteria. Forty-two full-text articles were assessed for eligibility, and 16 were excluded because they were non-clinical studies, reviews, non-English full texts, did not evaluate Tα1 as a major intervention, or met other exclusion criteria. Ultimately, 26 clinical publications were included in this scoping review (Figure 1).

### 3.2. Characteristics of Included Studies

#### 3.2.1. Study Design and Geographic Distribution

The 26 included publications comprised 22 involving classical Tα1, 3 involving TNF-α–Tα1 fusion proteins [26,27,28], and 1 involving mixed Tα1/thymopentin exposure [17] across diverse cancer populations and treatment settings. Most studies were conducted in China (21 studies, 80.8%), followed by Russia (3 studies, 11.5%), Italy (1 study, 3.8%), and South Korea (1 study, 3.8%). This distribution indicates that clinical research on Tα1 in cancer care has been geographically concentrated, particularly in China.

Retrospective observational studies were the most common design, accounting for 12 studies (46.2%), followed by 8 case reports (30.8%), 4 non-randomized clinical studies (15.4%), 1 prospective observational study (3.8%), and 1 randomized controlled trial (3.8%). Overall, the mapped evidence base relies mainly on retrospective and exploratory designs, whereas prospective randomized evidence remains limited. Detailed study-level characteristics are presented in Table 1. Among the advanced solid-tumor reports, one retrospective study evaluated Tα1 loading before immunoradiotherapy [29], whereas a multicenter phase II trial evaluated Tα1 with radiotherapy, camrelizumab, and GM-CSF [22]. These differences distinguish the reports but do not establish the absence of participant overlap.

#### 3.2.2. Cancer Type and Clinical Focus

The included studies covered a broad range of malignancies. Hepatobiliary cancers represented the largest disease cluster, accounting for 7 studies (26.9%), followed by thoracic cancers with 5 studies (19.2%) and gastrointestinal cancers with 4 studies (15.4%). Gynecologic cancers and multiple solid tumors were each evaluated in 2 studies (7.7%). Single studies were identified for central nervous system tumors, breast cancer, head and neck cancer, genitourinary cancer, skin and soft tissue malignancy, and hematologic or lymphoid malignancy.

The clinical contexts were also diverse, including postoperative or post-resection adjuvant treatment, advanced or metastatic disease, recurrent disease, chemoradiotherapy-based treatment, targeted therapy, immune checkpoint inhibitor-based therapy, and integrative supportive care. These findings suggest that Tα1 has been investigated primarily as an adjunctive immunomodulatory strategy across multiple cancer treatment settings rather than as a stand-alone anticancer intervention (Table 1).

### 3.3. Details in Intervention Characteristics

#### 3.3.1. Route of Administration, Dosage, and Treatment Duration

Tα1 was administered predominantly by subcutaneous injection. Most studies described Tα1 as subcutaneous injection, s.c. injection, thymalfasin injection, or Zadaxin-based treatment. One case report used intramuscular Tα1 injection as part of an integrative cancer care regimen. Several case reports used a TNF-α–Tα1 fusion product with interferon-γ, whereas some retrospective studies did not explicitly specify the formulation.

The most frequently reported dose was 1.6 mg, which was used across postoperative, chemoradiotherapy, targeted therapy, immunotherapy, and supportive-care settings. Twice-weekly administration was the most common schedule, although once-weekly, three-times-weekly, daily, twice-daily, every-other-day, and loading-dose schedules were also reported. Treatment duration ranged from one-day or short loading-dose strategies to long-term postoperative or maintenance treatment lasting several months to years. Short-term administration generally aimed to support immune recovery or mitigate treatment-related toxicity, whereas prolonged postoperative administration aimed to reduce recurrence risk. Detailed intervention characteristics are summarized in Table 2.

#### 3.3.2. Combination Strategies and Comparator Patterns

Tα1 was generally used in combination with other cancer therapies or supportive interventions. Combination strategies included chemotherapy, radiotherapy, concurrent chemoradiotherapy, EGFR-TKIs, PD-1/PD-L1 inhibitors, anti-CTLA-4 therapy, lenvatinib, sintilimab, camrelizumab, serplulimab, GM-CSF, antiviral therapy, nutritional support, Chinese herbal medicine, Korean medicine, hyperthermia, and sirolimus plus Huaier granule.

Comparator strategies varied according to study design and clinical context. Comparative studies used surgery alone, liver resection alone, antiviral therapy alone, EGFR-TKI monotherapy, concurrent chemoradiotherapy alone, XELOX chemotherapy alone, PD-1/PD-L1 inhibitor plus chemotherapy without Tα1, lenvatinib plus sintilimab without Tα1, or tacrolimus-based regimens as comparators. In contrast, case reports and several single-arm studies had no comparator and described clinical course or pre–post changes. These differences in comparators and co-interventions limited direct comparison across studies (Table 2).

### 3.4. Outcome Domains and Reporting Patterns

Across the included studies, outcome measures were diverse and reflected the wide range of cancer types and treatment contexts in which the interventions were evaluated (Table 3). Tumor response outcomes included complete response, partial response, objective response rate, disease control rate, pathological complete response, major pathological response, abscopal response, and RECIST-based response assessment. For classical Tα1, these outcomes were reported in a case report of Wilms tumor, as well as in clinical studies of thymic epithelial tumors, metastatic esophageal squamous cell carcinoma, platinum-resistant ovarian cancer, advanced or refractory metastatic solid cancers, locally advanced gastric or gastroesophageal junction adenocarcinoma, unresectable hepatocellular carcinoma, and advanced metastatic solid tumors [19,20,22,29,31,33,36,43]. Survival and recurrence outcomes included progression-free survival, overall survival, disease-free survival, recurrence-free survival, recurrence rate, local recurrence, distant metastasis, and follow-up duration, and were reported in studies of non-small cell lung cancer, hepatocellular carcinoma, colorectal cancer, melanoma, ovarian cancer, and advanced solid tumors, as well as in a case of chronic active Epstein–Barr virus infection with NK-cell lymphoma and hemophagocytic lymphohistiocytosis [15,18,19,20,21,22,29,31,32,33,34,38,40,41,42,43,44]. TNF-α–Tα1 fusion-protein reports described tumor response in recurrent glioblastoma, HER2-positive breast cancer, and recurrent cervical cancer [26,27,28]. The mixed Tα1/thymopentin study reported tumor response and survival in advanced EGFR-mutant non-small cell lung cancer [17].

Immune and inflammatory outcomes were also recurring domains. Several studies evaluated lymphocyte counts, CD3+ T cells, CD4+ T cells, CD8+ T cells, CD4+/CD8+ ratio, NK-cell activity, neutrophil-to-lymphocyte ratio, C-reactive protein, IL-2, IL-6, IFN-γ, immunoglobulins, regulatory T cells, and other immune-related markers. In locally advanced non-small cell lung cancer treated with concurrent chemoradiotherapy, Tα1 was associated with lower rates of grade ≥ 2 radiation pneumonitis and grade 3–4 lymphopenia, as well as lower frequency of markedly elevated C-reactive protein [39]. In advanced EGFR-mutant non-small cell lung cancer, thymic peptides (Tα1 or thymopentin) combined with EGFR-TKI therapy were associated with preservation of T-cell subsets compared with EGFR-TKI monotherapy [17]. These results could not be attributed specifically to Tα1. In colorectal cancer, Tα1 combined with XELOX was associated with improved CD3+, CD4+, and CD4+/CD8+ ratio in one randomized study, while another comparative study reported increased IL-2 and IFN-γ and decreased IL-6 after Tα1-containing treatment [40,41]. In hepatocellular carcinoma-related studies, Tα1-containing strategies were associated with changes in postoperative neutrophil-to-lymphocyte ratio or immune-related profiles in resection, antiviral therapy, and transplantation settings [34,38,44]. Case-based and clinical studies also reported normalization of T-lymphocyte counts, increased NK-cell cytotoxic activity, increased CD8+ T-cell percentage or CD4+/CD8+ ratio, and changes in immunoglobulins and lymphocyte subsets in cervical cancer, esophageal cancer, ovarian cancer, advanced or refractory metastatic solid cancers, and post-transplant hepatocellular carcinoma [19,29,33,44]. Immune-related findings in recurrent cervical cancer were reported separately for a TNF-α–Tα1 fusion regimen [28]. Studies using radiotherapy, immune checkpoint inhibitors, GM-CSF, or Tα1 loading-dose strategies also reported lymphocyte recovery, immune activation, or inflammatory markers [21,22,29]. Nonstandardized immune and inflammatory biomarker assessment precluded quantitative cross-study synthesis of immune recovery.

Patient-centered and supportive-care outcomes were reported across several case-based and clinical studies, although instruments and reporting approaches were inconsistent. Quality of life, Karnofsky Performance Status, general well-being, appetite, oral pain, functional recovery, clinical stability, and treatment tolerance were described in studies of recurrent glioblastoma, breast cancer, recurrent cervical cancer, refractory oral mucosal ulcer, thymic epithelial tumors, primary hepatocellular carcinoma, and Wilms tumor [26,27,28,30,31,35,36]. In the refractory oral mucosal ulcer case, clinical recovery and symptom relief were reported, including ulcer healing, disappearance of oral pain, and recovery of eating and drinking ability after Tα1 administration [30]. In metastatic melanoma, sequential exposure to Tα1 and anti-CTLA-4 therapy was associated with longer survival in retrospective analyses, although selection and treatment-sequence effects limit causal interpretation [32]. In metastatic esophageal squamous cell carcinoma, SBRT plus Tα1 was evaluated for abscopal response, survival, and immune modulation, indicating that Tα1 has also been explored in radiotherapy-associated immune response contexts [33]. In a prospective neoadjuvant gastric or gastroesophageal junction adenocarcinoma study, thymalfasin was incorporated into serplulimab plus SOX treatment, with outcomes including pathological response, surgical feasibility, nodal clearance, downstaging, tumor response, survival or recurrence follow-up, and adverse events [43].

### 3.5. Safety Reporting and Adverse Events

Safety reporting was heterogeneous across the included studies, and the level of detail varied substantially by study design and treatment context (Table 3). The findings below distinguish classical Tα1 from fusion proteins and mixed thymic-peptide exposures. Adverse events occurring during combination treatment were not automatically attributed to Tα1. Several case reports and single-arm studies described Tα1-containing regimens as generally well tolerated. Among the separately classified TNF-α–Tα1 fusion-protein reports, one recurrent glioblastoma case reported only grade 1 flu-like syndrome without treatment adjustment or discontinuation [26]. Case reports involving breast cancer, recurrent cervical cancer, refractory oral mucosal ulcer after targeted immunotherapy for hepatocellular carcinoma, and primary hepatocellular carcinoma generally reported no obvious treatment-related toxicities or no unexpected adverse events, although interpretation was limited by uncontrolled designs and concomitant therapies [27,28,30,35].

Some comparative studies suggested that Tα1 may mitigate treatment-related immune suppression or hematologic toxicity. In locally advanced non-small cell lung cancer treated with concurrent chemoradiotherapy, Tα1 was associated with reduced lymphopenia and was reported to be well tolerated, with only mild local irritation in 3 patients and no treatment-related severe toxicities [39]. In advanced EGFR-mutant non-small cell lung cancer, mixed thymic-peptide exposure (Tα1 or thymopentin) combined with EGFR-TKI was not associated with a reported increase in common adverse events such as rash, diarrhea, or dry skin compared with EGFR-TKI monotherapy [17]. In resected non-small cell lung cancer and HBV-related hepatocellular carcinoma, no new safety signals or serious drug-related adverse events were reported in Tα1-treated patients [18,38]. In metastatic esophageal squamous cell carcinoma, leukocytopenia occurred in 3 patients (9.7%), including grade 2 in 2 patients and grade 3 in 1 patient [33]. In colorectal cancer, lower neutropenia was reported in the Tα1 group in a comparative study of Tα1 combined with XELOX [41]. In platinum-resistant recurrent ovarian cancer, the total adverse-event incidence was lower in the Tα1 group, mainly because of reduced leukopenia and neutropenia [19].

Safety signals requiring caution were also identified. A nasopharyngeal carcinoma case described severe multisystem immune-related toxicity involving the skin, liver, lungs, and coagulation system after combination treatment including sintilimab, chemotherapy, nimotuzumab, and Tα1, followed by clinical improvement after corticosteroid treatment [37]. The contribution of Tα1 could not be separated from that of the concomitant treatments. In a study of unresectable or recurrent thymic epithelial tumors, grade 3 pneumonitis occurred in 1 of 50 patients (2.0%) [31]. In advanced or refractory metastatic solid cancers, no grade 4–5 adverse events were reported, although two grade 3 events, myocarditis and encephalitis, occurred in 2 of 48 patients (4.2%) and were attributed by the original authors to immunotherapy [29]. In locally advanced gastric or gastroesophageal junction adenocarcinoma, any-grade adverse events occurred in 93.3% of patients, but immune-related adverse events occurred in 23.3% and were grade 1–2, and no grade 3–4 immune-related adverse events or treatment-related deaths were reported [43].

In unresectable hepatocellular carcinoma, no significant between-group differences were observed in grade 1–2, grade 3–4, or overall treatment-related adverse events [20]. In advanced metastatic solid tumors treated with hypofractionated radiotherapy, PD-1 inhibition, GM-CSF, and Tα1, 17 adverse reactions were reported, including 6 grade 3–4 events, but no grade 5 events occurred [22]. These figures represent reported events and should not be interpreted as numbers of affected patients. In unresectable locally advanced non-small cell lung cancer, grade ≥ 3 hematologic toxicities and grade ≥ 2 pneumonitis were reported in the overall cohort, while grade ≥ 2 radiation pneumonitis was lower with Tα1 exposure [21]. In hepatocellular carcinoma beyond UCSF criteria after liver transplantation, sirolimus, thymalfasin, and Huaier granule triple-combination therapy was described as well tolerated, with no severe drug-induced liver or kidney injury during follow-up [44]. Safety outcomes were not reported in several publications and absence of reporting should not be interpreted as absence of harm. Differences in safety ascertainment, denominators, treatment regimens, and attribution precluded estimation of a pooled Tα1-specific adverse-event rate. The available findings therefore do not establish overall tolerability across cancer settings.

## 4. Discussion

This scoping review mapped 26 clinical publications involving classical Tα1 and separately classified related interventions in cancer care and found a broad but uneven evidence landscape across malignancies, treatment settings, and study designs. Findings from fusion-protein and mixed thymic-peptide studies were not used to infer the efficacy or safety of classical Tα1. Rather than supporting a single disease-centered indication, the literature concentrated on contexts in which immune suppression, treatment tolerance, recurrence control, or multimodal treatment support were clinically relevant. Tα1 was rarely evaluated as a stand-alone anticancer therapy; instead, it was generally positioned as an adjunctive immunomodulatory strategy alongside surgery, chemotherapy, radiotherapy, targeted therapy, immune checkpoint inhibitors, antiviral therapy, or integrative supportive care. This pattern is consistent with the proposed biological role of Tα1 as an immune-restorative or immune-supportive agent rather than a direct cytotoxic anticancer intervention [6,7,8]. These findings argue against a uniform role for Tα1 across all cancer populations. Its potential clinical value is more likely to depend on baseline immune status, cancer type and stage, treatment-induced immune suppression, the therapeutic backbone, and the intended treatment objective. Accordingly, Tα1 may be better viewed as a context-dependent immunomodulatory adjunct than as a broadly applicable anticancer intervention.

The predominance of hepatobiliary, thoracic, and gastrointestinal cancers is clinically meaningful. In hepatocellular carcinoma, Tα1 was investigated mainly in postoperative, post-resection, antiviral, transplantation, or unresectable disease settings, where recurrence control, survival, immune balance, and treatment tolerance were important outcome priorities [15,20,34,38,44]. In non-small cell lung cancer, classical Tα1 was evaluated across distinct treatment contexts, including EGFR-TKI therapy, post-R0 resection adjuvant immunomodulation, concurrent chemoradiotherapy, and consolidative immunotherapy [18,21,39], whereas the EGFR-TKI study involved mixed Tα1/thymopentin exposure [17]. These contexts should not be interpreted as a homogeneous clinical category, because each implies different therapeutic rationales: postoperative studies focused on recurrence and survival, chemoradiotherapy studies emphasized lymphopenia and pneumonitis, and targeted or immunotherapy-based studies incorporated both tumor-control and immune-preservation outcomes. This variation supports the development of clearly defined disease- and treatment-specific indications rather than extrapolation of findings across heterogeneous cancer populations.

Repeated comparative research was most apparent in hepatocellular carcinoma, non-small cell lung cancer, and colorectal cancer. In hepatocellular carcinoma, Tα1 was evaluated after resection, with antiviral treatment, after transplantation, and in combination with targeted therapy and immune checkpoint blockade, although the treatment objectives differed substantially among these settings [15,20,34,38,44]. In non-small cell lung cancer, available studies addressed postoperative recurrence, EGFR-TKI-associated immune suppression, chemoradiotherapy-related lymphopenia, radiation pneumonitis, and eligibility for consolidative immunotherapy [17,18,21,39]. In colorectal cancer, comparative studies focused mainly on postoperative XELOX-based treatment, recurrence, immune indices, quality of life, and hematologic toxicity [40,41]. By contrast, evidence in glioblastoma, breast cancer, cervical cancer, nasopharyngeal carcinoma, Wilms tumor, thymic epithelial tumors, gastric or gastroesophageal junction adenocarcinoma, hematologic or lymphoid malignancy, and mixed solid-tumor populations remained largely exploratory, single-arm, or case-based. These patterns identify settings in which further comparative research may be feasible while distinguishing areas that require additional clinical characterization before treatment effects can be meaningfully evaluated.

A recurring theme across the included studies was the use of Tα1 to preserve or restore host immune function during cancer treatment. Several studies reported T-cell subsets, CD4+/CD8+ ratio, NK-cell activity, immunoglobulins, neutrophil-to-lymphocyte ratio, C-reactive protein, IL-6, IL-2, IFN-γ, or lymphopenia-related endpoints [17,19,21,29,39,40,41,44]. This outcome profile supports the interpretation that Tα1 has been studied primarily in relation to immune competence and treatment resilience. Mechanistically, this clinical pattern is plausible because experimental studies have suggested that Tα1 can modulate immune-cell phenotypes and tumor microenvironmental signaling, including reversal of M2-like tumor-associated macrophage polarization [10]. However, most clinical studies did not directly connect immune biomarker changes with validated clinical endpoints, so the relationship between immune modulation and clinical benefit remains an important area for future research. Recent immunotherapy studies have shown that pretreatment peripheral immune profiles and routinely available blood-test variables may contribute to the stratification of treatment outcomes, supporting further investigation of accessible immune markers in combination-treatment research [45,46]. Candidate variables for Tα1 studies may include baseline absolute lymphocyte count, treatment-induced lymphopenia, neutrophil-to-lymphocyte ratio, T-cell subsets, NK-cell activity, inflammatory markers, and longitudinal immune-recovery patterns. However, none of these variables has been prospectively validated as a predictive biomarker of Tα1-specific benefit, and their current associations should not be interpreted as evidence for biomarker-guided clinical use.

Recent studies suggest an emerging interest in combining Tα1 with immune checkpoint inhibitor-based or multimodal immunoradiotherapy regimens. Tα1 was evaluated with PD-1/PD-L1 inhibitors plus chemotherapy in platinum-resistant recurrent ovarian cancer, lenvatinib plus sintilimab in unresectable hepatocellular carcinoma, hypofractionated radiotherapy plus PD-1 inhibition and GM-CSF in advanced metastatic solid tumors, and concurrent chemoradiotherapy with consolidative immunotherapy in locally advanced non-small cell lung cancer [19,20,21,22]. These studies reflect a shift from earlier use of Tα1 as a general immune adjuvant toward more specific immuno-oncology combination strategies. Nevertheless, the evidence remains preliminary because these studies differed in cancer type, treatment backbone, timing of Tα1 administration, dose, duration, and outcome definitions. The recent evaluation of short-term loading doses, prolonged treatment during chemoradiotherapy, and combinations with targeted agents or immune checkpoint inhibitors suggests that the timing and intended function of Tα1 may be as important as the administered dose itself [20,21,22,29]. Future trials should therefore clarify not only whether Tα1 improves lymphocyte recovery, treatment tolerance, or antitumor response, but also which patients, treatment phases, and combination regimens are most likely to benefit. Biomarker-stratified or response-adaptive designs may help distinguish patients requiring short-term immune restoration from those who may benefit from sustained immunomodulatory support. Recent biomarker-matched combination studies also illustrate how genomic and immune information can be jointly applied to treatment selection, although such an approach has not yet been evaluated for Tα1 [47].

The intervention characteristics also showed substantial heterogeneity. Although subcutaneous administration of 1.6 mg was the most common pattern, dose schedules ranged from once weekly to three times weekly, daily, twice daily, every other day, and loading-dose approaches. Treatment duration ranged from very short-term exposure to long-term postoperative or maintenance therapy lasting months to years. This variability likely reflects differences in intended clinical role, such as short-term immune support during intensive treatment, prevention of chemoradiotherapy-related lymphopenia, postoperative recurrence suppression, or long-term immune maintenance. However, it also limits comparability across studies, and the available evidence is insufficient to determine an optimal dose, dosing frequency, or treatment duration for a defined cancer type and treatment setting. Pharmacokinetic and pharmacodynamic relationships were rarely characterized in the included studies, making it difficult to determine whether fixed-dose regimens produce comparable biological effects across patients with different immune profiles, disease burdens, and concomitant therapies. Standardized intervention reporting should therefore be accompanied by predefined pharmacodynamic sampling, longitudinal immune monitoring, and dose- or schedule-finding analyses that relate Tα1 exposure to both biological and clinical outcomes. Such information would help move Tα1 research from empirically selected schedules toward more individualized treatment strategies.

Safety findings should be interpreted cautiously. Many studies described Tα1-containing regimens as tolerable, and several comparative studies reported no increase in common adverse events or suggested lower rates of leukopenia, neutropenia, lymphopenia, or pneumonitis in Tα1-exposed groups [17,18,19,21,38,39,41]. However, adverse-event reporting was inconsistent, and Tα1 was frequently administered within complex multimodal regimens involving chemotherapy, radiotherapy, targeted therapy, immune checkpoint inhibitors, antiviral therapy, immunosuppression, or integrative supportive care. One case described severe multisystem immune-related toxicity after combination treatment with sintilimab, chemotherapy, nimotuzumab, and Tα1, raising concern about possible immune overactivation but not establishing Tα1-specific causality [37]. Because adverse events occurred within multimodal treatment regimens, attribution to Tα1 alone was frequently difficult. Future safety assessments should consider treatment sequence, temporal relationships, baseline immune or autoimmune risk, concomitant immunomodulatory agents, and serial changes in immune and inflammatory markers. Particular attention should be paid to whether Tα1 modifies the efficacy, immune recovery, or toxicity of chemotherapy, radiotherapy, targeted therapy, or immune checkpoint blockade. Therefore, future studies should include structured adverse-event reporting, causality assessment, and careful monitoring of immune-related adverse events, especially when Tα1 is combined with ICIs. Combination-specific monitoring plans and predefined stopping rules may be particularly important for patients with previous immune-related toxicity or evidence of excessive immune activation.

This review has several limitations. First, the evidence base was geographically concentrated, with most studies conducted in China, which may limit generalizability to other healthcare systems and oncology practice settings. Differences in disease etiology, background anticancer treatment, access to Tα1, and supportive-care practices may limit the transferability of these findings to other settings. Preferential publication of favorable findings may also have influenced the mapped evidence, although publication bias was not formally assessed. Second, the included studies were dominated by retrospective observational studies and case reports, while randomized controlled evidence was scarce. The publication-date restriction excluded earlier clinical studies, including randomized trials. Therefore, the mapped distribution of study designs and clinical findings does not represent the entire historical evidence base for Tα1 in oncology. Retrospective comparisons remain susceptible to selection bias and residual confounding, including after propensity-score matching. Case reports describe individual clinical courses and cannot establish comparative efficacy or the frequency of adverse events. Potential participant overlap between publications could not be definitively excluded. Publication counts should therefore not be interpreted as counts of independent patient cohorts. Third, substantial heterogeneity in cancer types, disease stages, treatment backbones, Tα1 exposure, comparators, and outcome measures prevented quantitative synthesis. Fourth, safety reporting was incomplete or inconsistently structured in several studies, limiting assessment of Tα1-specific harms. Fifth, eligibility was restricted to English-language publications, which may have resulted in language-related selection bias, particularly because Tα1 has been investigated in several non-English-speaking healthcare settings. Sixth, formal risk-of-bias and certainty-of-evidence assessments were not performed because the review was designed to map the available evidence rather than estimate treatment effects. Accordingly, favorable findings from individual studies should not be interpreted as definitive evidence of efficacy. Finally, because this review focused on published clinical studies, it may not fully capture unpublished findings, ongoing trials, or preclinical evidence relevant to mechanisms, pharmacodynamics, and treatment interactions.

Despite these limitations, this review provides a clinically oriented map of how Tα1 has been investigated in cancer care. The findings suggest that priority areas for future research may include postoperative or post-resection recurrence prevention in hepatocellular carcinoma and non-small cell lung cancer, mitigation of lymphopenia during chemoradiotherapy, immune-supportive use in EGFR-TKI or ICI-based regimens, and multimodal immunoradiotherapy strategies in advanced solid tumors. Postoperative trials could prioritize recurrence-free or disease-free survival, with overall survival follow-up. In non-small cell lung cancer treated with chemoradiotherapy, trials could prioritize a prespecified clinical toxicity endpoint, such as grade ≥ 2 radiation pneumonitis, together with treatment completion. Future studies should use prospectively defined PICO frameworks, standardized dosing and timing protocols, clinically meaningful comparators, harmonized immune and oncologic outcome domains, systematic harms reporting, and prespecified pharmacodynamic assessments. Randomized comparisons should use the same background anticancer regimen in both groups to help isolate the contribution of Tα1. They should also incorporate longitudinal immune monitoring and carefully planned subgroup analyses based on baseline immune status, inflammatory burden, treatment-induced lymphopenia, and concomitant anticancer therapy. Candidate biomarkers should be evaluated prospectively and linked to clearly defined clinical outcomes before being used for patient selection. Such work will be necessary to clarify the clinical settings and patient populations in which Tα1 may provide benefit.

## 5. Conclusions

This scoping review mapped the current clinical evidence on Tα1 in cancer care and found that Tα1 has been investigated mainly as an adjunctive immunomodulatory strategy across diverse malignancies and treatment settings. The evidence was concentrated in hepatobiliary, thoracic, and gastrointestinal cancers, with most studies conducted in China and dominated by retrospective observational studies and case reports. Tα1 was administered predominantly by injection, most commonly at 1.6 mg, and was used in combination with surgery, chemotherapy, radiotherapy, targeted therapy, immune checkpoint inhibitors, antiviral therapy, or supportive and integrative care. Reported outcomes included tumor response, survival, recurrence, immune and inflammatory markers, treatment-related toxicities, functional status, quality of life, and safety findings. Although several studies reported favorable clinical, immune-related, or safety signals, the evidence remains heterogeneous in terms of cancer type, treatment context, dosing schedule, comparator, and outcome reporting. Future prospective controlled studies with standardized Tα1 regimens, clearly defined clinical indications, harmonized outcome domains, and systematic adverse-event assessment are needed to clarify the role of Tα1 as an evidence-based adjunctive immunomodulatory strategy in cancer care. Trials involving immune checkpoint inhibitors should additionally include prospective immune-related adverse-event monitoring and predefined stopping criteria.

## Figures and Tables

**Table 1:** Characteristics of included clinical studies on thymosin alpha 1.

Study ID	Country	Study Design	Disease/Condition	System Category	Sample Size	Age
Alyasova 2024 [26]	Russia	Case report	Recurrent Glioblastoma (GBM)	Central nervous system	1	46
Ben 2025 [27]	Russia	Case report	HER2/neu-positive breast cancer (Stage III, cT2N3M0)	Breast	1	47
Bykova 2024 [28]	Russia	Case report	Recurrent cervical cancer (Stage IIIB)	Gynecologic	1	37
Chai 2025 [30]	China	Case report	Refractory oral mucosal ulcer after targeted immunotherapy for HCC	Hepatobiliary	1	65
Chu 2022 [31]	China	Non-RCT	Unresectable or recurrent thymic epithelial tumor (TET)	Thoracic	50	49 (30–66)
Danielli 2018 [32]	Italy	Retrospective observational study	Metastatic melanoma (MM)	Skin and soft tissue	Tα1 cohort (61) [IPI+: 21/IPI-: 40] IPI cohort (95) [Tα1+: 17/Tα1-: 78]	61 (14–85)
Du 2018 [33]	China	Prospective observational study	Metastatic esophageal squamous cell carcinoma	Gastrointestinal	31	59 (46–71)
Feng 2021 [17]	China	Retrospective observational study	Advanced NSCLC with active EGFR mutations (Exon 19 del or L858R)	Thoracic	TG: 65/CG: 267 (332) [matched: TG: 65/CG: 130 (195)]	57.5 (21–81)
Guo 2021 [18]	China	Retrospective observational study	Stage IA-IIIA Non-Small Cell Lung Cancer (NSCLC) after R0 resection	Thoracic	TG: 1027/CG: 4719 (5746) [matched: TG: 1027/CG: 1027]	TG: median 59 (31–76) CG: 59 (33–77)
He 2017 [34]	China	Retrospective observational study	Small Hepatocellular Carcinoma (HCC) after liver resection	Hepatobiliary	TG: 44/CG: 162 (206)	TG: 49.8 ± 11.9 CG: 51.7 ± 11.6
Hu 2023 [35]	China	Case report	Primary hepatocellular carcinoma (PLC)	Hepatobiliary	1	104
Lee 2016 [36]	South Korea	Case report	Stage IV Wilms Tumor (Meta to liver, lung)	Genitourinary	1	17
Li 2026 [37]	China	Case report	Stage cT4N3M0 nasopharyngeal carcinoma	Head and neck	1	29
Liang 2016 [15]	China	Retrospective controlled study	HBV-associated HCC after radical hepatectomy	Hepatobiliary	TG: 146/CG: 412 (558). Matched TG: 106/CG: 106 (212)	Before matching, TG 49.8 ± 9.9/CG 50.7 ± 11.6. After matching, TG 49.0 ± 10.1/CG 49.7 ± 10.8
Linye 2021 [38]	China	Retrospective observational study	Solitary HBV-related hepatocellular carcinoma (HCC) after curative resection	Hepatobiliary	TG: 228/CG: 240 (468) [matched: TG: 100/CG: 100]	Before Matching: 52.8 ± 11.7 (CG)/49.7 ± 13.2 (TG) After Matching: 52.8 ± 11.7 (CG)/39.6 ± 11.0 (TG)
Liu 2022 [39]	China	Non-RCT	Unresectable locally advanced non-small cell lung cancer (LA-NSCLC)	Thoracic	TG: 69/CG: 69 (138)	TG: Median 58 (29–73) CG: Median 57 (34–71)
Niu 2024 [40]	China	RCT	Colorectal cancer (CRC) after radical resection	Gastrointestinal	TG: 199/CG: 201 (400)	TG: 64.88 ± 16.26 High TG: 64.44 ± 17.50 CG: 65.52 ± 17.15
Sha 2024 [41]	China	Retrospective observational study	Colorectal cancer (CRC) after radical surgery	Gastrointestinal	TG: 94/CG: 86 (180)	TG: 55.39 ± 6.99 CG: 54.49 ± 7.67
Wang 2019 [42]	China	Case report	Chronic active EBV infection (CAEBV) with NK cell lymphoma and HLH	Hematologic and lymphoid	1	27
Wang 2025 [19]	China	Retrospective observational study	Platinum-resistant recurrent ovarian cancer (PROC)	Gynecological	TG: 193/CG: 193 (386)	TG: 50.1 ± 8.8 CG: 49.9 ± 9.2
Xu 2025 [29]	China	Retrospective observational study	Advanced or refractory metastatic solid cancers	Multiple solid tumors	1.6 mg: 17/3.2 mg: 31	Median 61 (36–87)
Xu 2026 [43]	China	Non-RCT	Locally advanced gastric or gastroesophageal junction adenocarcinoma	Gastrointestinal	30	Median 65.5 (60.0–70.8)
Yao 2025 [20]	China	Retrospective observational study	Unresectable hepatocellular carcinoma (uHCC)	Hepatobiliary	TG: 43/CG: 49 (92)	Mean: 53.3 ± 11.8 TG: 51.9 ± 10.8 CG: 54.5 ± 12.7
Yu 2025 [22]	China	Non-RCT	Heavily treated metastatic solid tumors	Multiple solid tumors	37 enrolled/30 ITT/26 efficacy-evaluable	Median 64 (36–74)
Zhang 2025 [21]	China	Retrospective observational study	Unresectable stage IIIA-IIIC LA-NSCLC	Thoracic	TG: 148 (101 short term, 47 long term)/CG: 48 (196)	Median: 59.5 (IQR: 54–65) TG (long): 58 (53–67) TG (short): 58 (54–65) CG: 62.5 (56–66)
Zhou 2018 [44]	China	Retrospective observational study	HCC beyond UCSF criteria after Liver Transplantation	Hepatobiliary	TG: 18/CG: 18 (36)	TG: 53.94 ± 7.40 CG: 49.72 ± 7.00

RCT, randomized controlled trial; GBM, glioblastoma; HCC, hepatocellular carcinoma; TET, thymic epithelial tumor; MM, metastatic melanoma; NSCLC, non-small cell lung cancer; EGFR, epidermal growth factor receptor; del, deletion; PLC, primary liver cancer; HBV, hepatitis B virus; LA-NSCLC, locally advanced non-small cell lung cancer; CRC, colorectal cancer; CAEBV, chronic active Epstein–Barr virus infection; NK, natural killer; HLH, hemophagocytic lymphohistiocytosis; PROC, platinum-resistant recurrent ovarian cancer; uHCC, unresectable hepatocellular carcinoma; UCSF, University of California, San Francisco; TG, treatment group; CG, control group; IPI, ipilimumab; Tα1, thymosin alpha 1; IQR, interquartile range.

**Table 2:** Characteristics of thymosin alpha 1 interventions across included clinical studies.

Study ID	Intervention	Formulation	Dosage	Frequency	Treatment Duration	Comparator
Alyasova 2024 [26]	Intra-arterial bevacizumab followed by TNF-α–Tα1 plus recombinant IFN-γ	Subcutaneous injection	TNF-α–Tα1 100,000 IU; recombinant IFN-γ 500,000 IU	Every other day	9 courses; 20 days per course	None
Ben 2025 [27]	Standard neoadjuvant therapy with paclitaxel, trastuzumab, and pertuzumab plus recombinant IFN-γ and TNF-α–Tα1	Subcutaneous injection	TNF-α–Tα1 100,000 IU	Every other day	8 courses; every other day × 10 administrations per course, over 5–6 months	None
Bykova 2024 [28]	Refnot (TNF-α–Tα1) plus Ingaron (IFN-γ) and chemotherapy with cisplatin plus gemcitabine	Subcutaneous injection	TNF-α–Tα1 100,000 IU	Once daily	6 cycles of polychemotherapy plus ongoing maintenance therapy for >28 months	None
Chai 2025 [30]	Tα1 plus nutritional agent and prednisone acetate	Subcutaneous injection	1.6 mg	Twice daily	3 weeks	None
Chu 2022 [31]	Hypofractionated RT plus docetaxel, nedaplatin, and Tα1	Subcutaneous injection	1.6 mg	Once weekly	From the start of RT to 2 months after RT completion; median 14 weeks	None
Danielli 2018 [32]	Tα1 plus dacarbazine, followed by anti-CTLA-4 therapy with ipilimumab or tremelimumab	NR	NR	NR	NR	Tα1 cohort: Tα1 without anti-CTLA-4 therapy; IPI cohort: anti-CTLA-4 therapy without prior Tα1
Du 2018 [33]	SBRT plus Tα1	Subcutaneous injection	1.6 mg	Twice weekly	SBRT for 1 week; Tα1 until documented progression of non-irradiated metastatic lesions	None
Feng 2021 [17]	EGFR-TKI plus thymic peptide (Tα1 or thymopentin [TP5])	NR	Tα1 1.6 mg; TP5 10 mg	Twice weekly (Tα1); once daily (TP5)	At least 4 weeks	EGFR-TKI monotherapy
Guo 2021 [18]	Tα1 plus postoperative standard care	Subcutaneous injection	1.6 mg	Twice weekly	4–159 months; median 20 months; initiated 1–3 months after surgery	Postoperative standard care without Tα1 after R0 resection
He 2017 [34]	Liver resection plus Tα1	Subcutaneous injection	1.6 mg	Twice weekly	At least 26 weeks	Liver resection without Tα1
Hu 2023 [35]	Tα1 plus Chinese herbal medicine, nutritional support, and treatment for comorbidities	NR	1.6 mg	Twice weekly	NR	None
Lee 2016 [36]	Tα1 (Zadaxin) plus Korean medicine and hyperthermia	Intramuscular injection	1.6 mg	2–3 times weekly	9 months	None
Li 2026 [37]	Sintilimab plus paclitaxel, nedaplatin, nimotuzumab, and Tα1	Subcutaneous injection	1.6 mg	Once daily	1 day	None
Liang 2016 [15]	Radical hepatectomy plus Tα1	Subcutaneous injection	1.6 mg	Twice weekly	6 months	Radical hepatectomy without Tα1
Linye 2021 [38]	Tα1 plus antiviral therapy with ETV, TDF, or ADV	Subcutaneous injection	1.6 mg	Twice weekly	At least 6 months	Antiviral therapy without Tα1
Liu 2022 [39]	Tα1 plus CCRT	Subcutaneous injection	1.6 mg	Once weekly	From the start of CCRT to 2 months after CCRT completion	CCRT without Tα1
Niu 2024 [40]	Tα1 plus XELOX	Subcutaneous injection	1.6 mg	Group A: twice weekly; Group B: three times weekly	Group A: until 6 months after surgery; Group B: from 1 day before surgery to 6 months after surgery	XELOX without Tα1
Sha 2024 [41]	Tα1 plus XELOX	Subcutaneous injection	1.6 mg	Three times weekly	4 cycles of chemotherapy; approximately 12 weeks	XELOX without Tα1
Wang 2019 [42]	Symptomatic treatment plus antiviral therapy with acyclovir and foscarnet sodium, and adjunctive treatment with Tα1 and methylprednisolone	Subcutaneous injection	NR	NR	42 days	None
Wang 2025 [19]	Tα1 plus PD-1/PD-L1 inhibitor and chemotherapy	Subcutaneous injection	1.6 mg	Three times weekly	Until disease progression, unacceptable toxicity, or withdrawal	PD-1/PD-L1 inhibitor plus chemotherapy without Tα1
Xu 2025 [29]	Tα1 loading dose plus hypofractionated RT and PD-1 inhibitor	Subcutaneous injection	1.6 mg or 3.2 mg	Once daily	7 consecutive days as loading dose	None; pre–post evaluation and dose-group comparison
Xu 2026 [43]	Neoadjuvant serplulimab plus SOX and Tα1	Subcutaneous injection	4.8 mg	Twice weekly	9 weeks	None
Yao 2025 [20]	Tα1 plus lenvatinib and sintilimab	Subcutaneous injection	1.6 mg	Twice weekly	Until unacceptable toxicity or death; continuation beyond PD allowed if clinical benefit was present	Lenvatinib plus sintilimab without Tα1
Yu 2025 [22]	HFRT plus camrelizumab, GM-CSF, and Tα1	Subcutaneous injection	1.6 mg	Twice weekly	Until disease progression or study endpoint	None
Zhang 2025 [21]	CCRT with docetaxel, cisplatin, and radiotherapy ± nivolumab plus Tα1	Subcutaneous injection	1.6 mg	Once weekly	Short-term Tα1: during CCRT; long-term Tα1: during CCRT and up to 12 months after CCRT	Comparison among non-Tα1, short-term Tα1, and long-term Tα1 groups
Zhou 2018 [44]	Sirolimus plus thymalfasin and Huaier granule	Subcutaneous injection for thymalfasin; oral administration for sirolimus and Huaier granule	1.6 mg	Once daily for 10 days, then twice weekly	From 1 week after LT to the end of follow-up	Tacrolimus-based regimen

ADV, adefovir; CCRT, concurrent chemoradiotherapy; CTLA-4, cytotoxic T-lymphocyte-associated protein 4; EGFR-TKI, epidermal growth factor receptor tyrosine kinase inhibitor; ETV, entecavir; GM-CSF, granulocyte-macrophage colony-stimulating factor; HFRT, hypofractionated radiotherapy; IFN-γ, interferon gamma; IPI, ipilimumab; LT, liver transplantation; NR, not reported; PD, progressive disease; PD-1, programmed cell death protein 1; PD-L1, programmed death-ligand 1; RT, radiotherapy; SBRT, stereotactic body radiotherapy; SOX, S-1 plus oxaliplatin; TDF, tenofovir disoproxil fumarate; TNF-α–Tα1, tumor necrosis factor alpha–thymosin alpha 1 fusion protein; TP5, thymopentin; Tα1, thymosin alpha 1; XELOX, capecitabine plus oxaliplatin.

**Table 3:** Outcome Measures, Main Findings, and Safety Reporting of Included Studies.

Study ID	Outcome Measures	Main Findings	Safety (AE)
Alyasova 2024 [26]	1. Tumor response; 2. QOL/performance status; 3. survival; 4. biomarkers	1. Complete response (CR) was achieved, confirmed by contrast-enhanced MRI and PET imaging; 2. KPS improved from 80% to 90% during treatment; 3. overall survival (OS) exceeded 25 months, longer than the usual prognosis for recurrent GBM (<17 months); 4. serum TNF-α1 levels increased and stabilized during cytokine therapy courses.	Cytokine therapy was well tolerated. Grade 1 flu-like syndrome was reported according to CTCAE 5.0, without treatment adjustment or discontinuation.
Ben 2025 [27]	1. Tumor response; 2. QOL/performance status; 3. biomarkers	1. Pathological complete response (pCR) with residual cancer burden (RCB) 0 was achieved after 8 cycles, with complete regression in the breast and axillary/supraclavicular lymph nodes; 2. well-being improved after two cycles, with nausea reduced to grade 1, no vomiting, and restored appetite; 3. KPS increased from 70% to 95%, and blood TNF-α1 rose from <1.0 pg/mL to 30.6 pg/mL.	Treatment was well tolerated; no side effects specifically associated with TNF-α–Tα1 or IFN-γ were reported.
Bykova 2024 [28]	1. Tumor response; 2. immune modulation; 3. QOL/performance status	1. CR was achieved according to RECIST 1.1 after 6 cycles of combined polychemotherapy and cytokine therapy, and persisted for 28 months after completion of polychemotherapy; 2. absolute and relative T-lymphocyte counts normalized, and NK-cell cytotoxic activity increased; 3. QOL and functional status improved.	Treatment was well tolerated; no clinically significant side effects or toxicities were reported.
Chai 2025 [30]	1. Clinical recovery; 2. symptom relief; 3. mechanistic findings	1. Extensive oral mucosal ulcers and hemorrhage completely healed within 3 weeks after addition of Tα1; 2. oral pain disappeared, and the patient regained the ability to eat and drink normally; 3. Tα1 was suggested to regulate the immune microenvironment and promote T-lymphocyte maturation.	No obvious adverse reactions or drug-related toxicities were observed during 3 weeks of Tα1 administration.
Chu 2022 [31]	1. Tumor response; 2. survival; 3. QOL; 4. immune status	1. ORR was 83.7% across all dose levels; 2. the 2-year PFS rate was 59.1% (95% CI, 51.1–67.1%), and the 2-year OS rate was 90.0% (95% CI, 85.2–94.8%); 3. most patients (88.0%) maintained stable health-related QOL within 12 months after RT; 4. persistent lymphopenia at 6 months post-RT was observed in 51.3% of patients.	Grade 3 pneumonitis occurred in 1 patient (2.0%). No other grade ≥ 3 non-hematologic toxic effects were reported.
Danielli 2018 [32]	1. Overall survival; 2. long-term survival; 3. sequential immunotherapy outcomes	1. Median OS was 57.8 months in patients treated sequentially with Tα1 and anti-CTLA-4 therapy vs. 7.4 months in the Tα1-alone group (*p* < 0.0001); 2. in the ipilimumab cohort, patients pretreated with Tα1 had median OS of 38.4 months vs. 8.0 months in those never exposed to Tα1 (*p* = 0.03); 3. the 5-year OS rate was 41.2% in the Tα1–IPI sequence group vs. 13.0% in the IPI-only group (*p* = 0.006).	NR
Du 2018 [33]	1. Tumor response/abscopal effect; 2. survival; 3. immune modulation	1. Disease control in non-irradiated metastatic lesions was observed in 14/31 patients (45.2%), including PR in 3 patients (9.7%) and SD in 11 patients (35.5%); 2. median APFS was 2.9 months, and median OS was 5.2 months; 3. the proportion of CD8+ T cells after treatment was higher in patients with abscopal disease control, whereas no significant overall changes in lymphocyte subsets were observed.	No grade 4 acute toxicity was observed. Leukocytopenia occurred in 3 patients (9.7%), including grade 2 in 2 patients and grade 3 in 1 patient.
Feng 2021 [17]	1. PFS; 2. OS; 3. Immune preservation; 4. tumor response	1. Median PFS was 14.4 months in the combination group vs. 9.2 months in the TKI monotherapy group (*p* < 0.0001); 2. Median OS was 29.5 months vs. 19.8 months (*p* < 0.0001); 3. TKI monotherapy reduced CD3+, CD4+, and CD8+ T cells, whereas the thymic-peptide combination reversed this inhibition; 4. ORR was 60.0% vs. 60.8%, and DCR was 96.9% vs. 97.7%, respectively.	The combination did not increase common AEs such as rash, diarrhea, or dry skin compared with EGFR-TKI monotherapy.
Guo 2021 [18]	1. OS; 2. DFS; 3. stage-specific survival; 4. prognostic factors	1. After PSM, the 5-year OS rate was 82.1% in the Tα1 group vs. 78.4% in controls (*p* = 0.017); 2. the 5-year DFS rate was 77.3% vs. 63.8% (*p* < 0.001); 3. survival benefits were particularly significant in stage II and III disease; 4. multivariate analysis identified Tα1 as an independent protective factor for both OS and DFS.	No drug-related serious AE affecting survival, no AE leading to discontinuation, and no new safety signals were reported.
He 2017 [34]	1. OS; 2. RFS; 3. NLR modulation; 4. prognostic factors	1. The 5-year OS rate was 82.9% in the Tα1 group vs. 62.9% in controls (*p* = 0.014); 2. the 5-year RFS rate was 53.3% vs. 32.1% (*p* = 0.015); 3. Tα1 reduced postoperative NLR through lymphocyte increase and neutrophil decrease; 4. multivariate analysis identified Tα1 as an independent prognostic factor for OS and RFS.	NR
Hu 2023 [35]	1. QOL; 2. tumor management; 3. psychological status	1. High QOL was maintained, with preserved daily functioning such as face washing, eating, reading, and note-taking; 2. tumor size increased from 2.32 × 2.84 cm to 4.00 × 5.05 cm, but the patient remained asymptomatic and clinically stable for 27 months; 3. MMSE remained 27, with no anxiety or depression.	Good compliance and tolerance were reported, without drug-related complications or unexpected events.
Lee 2016 [36]	1. Clinical efficacy; 2. tumor response; 3. immune support	1. Clinical management was achieved in a patient who failed or refused conventional chemotherapy; 2. liver metastasis regressed, and lung metastasis remained stable; 3. Tα1 was used as a key immunomodulator in combination with Korean medicine.	NR
Li 2026 [37]	1. Immune-related toxicity; 2. symptom course; 3. recovery; 4. rechallenge outcome	1. Severe multisystem irAE developed involving the skin, liver, lungs, and coagulation system; 2. manifestations included high fever (39.5–40.5 °C), diffuse rash, interstitial pulmonary edema, and alveolar effusion; 3. symptoms resolved rapidly after intravenous methylprednisolone 2 mg/kg daily for 12 days; 4. sintilimab rechallenge 3–5 months later without Tα1 did not result in recurrent severe irAE.	Severe multisystem immune-related toxicity was reported after combination treatment including sintilimab and Tα1, requiring urgent corticosteroid treatment.
Liang 2016 [15]	1. OS; 2. RFS	1. After matching, 1-, 2-, and 3-year OS rates were 87.2%, 82.0%, and 68.4% in the Tα1 group versus 78.2%, 64.2%, and 49.7% in controls (*p* = 0.011); 2. corresponding RFS rates were 79.7%, 70.8%, and 67.3% versus 69.9%, 61.5%, and 51.6% (*p* = 0.019).	NR
Linye 2021 [38]	1. OS; 2. RFS; 3. immune response	1. After PSM, the 5-year OS rate was 55.5% in the Tα1 group vs. 47.2% in controls (*p* < 0.001); 2. the 5-year RFS rate was 58.2% vs. 32.6% (*p* = 0.006); 3. postoperative NLR was significantly lower in the Tα1 group (*p* < 0.001).	No serious drug-related SAE was reported during Tα1 treatment.
Liu 2022 [39]	1. Lymphopenia mitigation; 2. anti-inflammatory effects; 3. clinical treatment tolerance	1. The incidence of grade ≥ 2 radiation pneumonitis was 36.2% in the Tα1 group vs. 53.6% in controls (*p* = 0.040); 2. grade 3–4 lymphopenia occurred in 19.1% vs. 62.1% (*p* < 0.001); 3. CRP > 100 mg/L was less frequent in the Tα1 group (13.8% vs. 29.7%, *p* = 0.029); 4. Tα1 helped maintain total lymphocyte count during the most intensive phase of treatment.	Treatment was well tolerated; only mild local irritation was reported in 3 patients, and no treatment-related severe toxicities were observed.
Niu 2024 [40]	1. DFS; 2. perioperative immune function; 3. postoperative complications	1. Median DFS was 29.0 months in the experimental group vs. 22.0 months in controls (*p* < 0.001); local recurrence occurred in 3.00% vs. 7.50% (*p* < 0.05), and distant metastasis in 2.50% vs. 7.50% (*p* < 0.05); 2. CD3+ T cells, CD4+ T cells, and CD4+/CD8+ ratio were higher in the Tα1 group (all *p* < 0.001); 3. early complications were 3.50% vs. 9.50% (*p* < 0.05), and late complications were 4.50% vs. 10.00% (*p* < 0.05).	NR
Sha 2024 [41]	1. Recurrence control; 2. immune and inflammatory modulation; 3. tumor markers; 4. QOL	1. The 1-year recurrence rate was similar between groups, but the 3-year recurrence rate was 24.47% in the Tα1 group vs. 59.30% in controls (*p* < 0.001); 2. CD3+, CD4+, and CD4+/CD8+ ratio increased, IL-2 and IFN-γ increased, and IL-6 decreased (all *p* < 0.05); 3. serum tumor markers CEA, CA242, and CA724 decreased more in the Tα1 group (*p* < 0.001); 4. physical, role, cognitive, emotional, and social function scores improved significantly (*p* < 0.001).	Neutropenia occurred less frequently in the Tα1 group (32.98% vs. 48.84%, *p* = 0.030).
Wang 2019 [42]	1. Clinical presentation and diagnosis; 2. treatment response and disease progression; 3. survival	1. The patient initially presented with mild splenomegaly and recurrent liver dysfunction and was initially misdiagnosed as drug-induced liver injury; 2. antiviral therapy and adjunctive Tα1 plus methylprednisolone were administered; 3. the patient died of multiple organ failure 42 days after admission.	NR
Wang 2025 [19]	1. Survival and tumor response; 2. immune function; 3. hematologic toxicity/myelosuppression	1. ORR was 43.0% in the Tα1 group vs. 30.1% in controls (*p* = 0.009), and DCR was 87.0% vs. 69.9% (*p* < 0.001); 2. IgA, IgG, IgM, CD3+, CD4+, NK cells, and CD4+/CD8+ ratio all increased after treatment (all *p* < 0.01); 3. Leukopenia was 27.98% vs. 48.78%, and neutropenia was 18.65% vs. 37.64% (both *p* < 0.001).	The total AE incidence was lower in the Tα1 group (50.8% vs. 65.8%, *p* = 0.004), mainly due to reduced leukopenia and neutropenia.
Xu 2025 [29]	1. Lymphocyte subset changes; 2. survival; 3. tumor response	1. Median CD3+ T cells increased from 422.5 to 614.0/μL, CD4+ T cells from 244.5 to 284.5/μL, and CD8+ T cells from 159.0 to 222.5/μL after 7 days (all *p* < 0.001); 2. median PFS was 5.1 months (95% CI, 2.97–8.0), and median OS was 9.6 months (95% CI, 6.33–19.67); 3. among 36 evaluable patients, ORR was 19.4%, and DCR was 69.4%.	No grade 4–5 AEs were reported. Two patients (4.2%) had grade 3 events, namely myocarditis and encephalitis, considered related to immunotherapy.
Xu 2026 [43]	1. Pathological response; 2. nodal clearance and downstaging; 3. survival and recurrence; 4. tumor response	1. The pCR rate was 30.0% (9/30), and the MPR rate was 56.7% (17/30); 2. ypN0 was achieved in 63.3% (19/30), and overall N-stage downstaging in 80.0% (24/30); 3. at a median follow-up of 14.0 months, no deaths were documented, and only 1 retroperitoneal nodal relapse occurred; 4. ORR was 73.3%, and DCR was 100% by RECIST 1.1.	Any-grade AEs occurred in 93.3% of patients; irAEs occurred in 23.3%, all grade 1–2. No grade 3–4 irAEs or treatment-related deaths were reported.
Yao 2025 [20]	1. OS; 2. PFS; 3. ORR; 4. DCR	1. Median OS was 16.0 months in the experimental group vs. 11.0 months in controls (*p* = 0.018); 2. median PFS was 7.0 months vs. 4.0 months (*p* = 0.006); 3. ORR was 55.8% vs. 34.7% (*p* = 0.042); 4. DCR was 76.7% vs. 59.2%, but the difference was not statistically significant (*p* = 0.073).	No significant between-group differences were observed in grade 1–2, grade 3–4, or overall treatment-related AEs (*p* > 0.05).
Yu 2025 [22]	1. PFS; 2. tumor response; 3. abscopal effect; 4. prognostic marker	1. Median PFS in the ITT population was 3.50 months (95% CI, 2.77–4.23); 2. ORR was 23.08%, and DCR was 65.38% according to RECIST 1.1; 3. abscopal effects were observed in 6 patients (23.08%) and were associated with partial response (*p* = 0.025); 4. baseline NLR < 5 was associated with lower risk of distant metastasis and death (*p* = 0.024).	A total of 17 adverse reactions were reported, including 6 grade 3–4 events such as thrombocytopenia and leukopenia. No grade 5 events occurred.
Zhang 2025 [21]	1. PFS; 2. OS; 3. eligibility for consolidative immunotherapy; 4. treatment-related pneumonitis; 5. lymphocyte recovery; 6. IL-6	1. Median PFS was 14.6 months in the non-Tα1 group, 16.0 months in the short-term Tα1 group, and not reached in the long-term Tα1 group; long-term Tα1 significantly improved PFS vs. non-Tα1 (HR = 0.544, *p* = 0.03); 2. median OS was 20.0 months, 27.6 months, and not reached, respectively; long-term Tα1 improved OS (HR = 0.402, *p* = 0.01); 3. eligibility for consolidative nivolumab was 93.6% in the long-term Tα1 group vs. 75.2% in the short-term Tα1 group and 77.1% in the non-Tα1 group (*p* = 0.03); 4. grade ≥ 2 pneumonitis was 14.5% in the long-term Tα1 group vs. 35.4% in the non-Tα1 group (*p* = 0.02); 5. lymphopenia at 6 months post-CCRT was 22.5% vs. 30.9% vs. 55.8% (*p* = 0.01); 6. median IL-6 at 2 months post-CCRT was 4.92 pg/mL in the long-term Tα1 group vs. 8.14 pg/mL in the non-Tα1 group (*p* = 0.03).	The most common grade ≥ 3 hematologic toxicity was lymphopenia (118/196, 60.2%), followed by neutropenia (26/196, 13.3%). Grade ≥ 2 pneumonitis occurred in 49/196 patients (25.0%). Radiation pneumonitis occurred in 9/48 (18.8%), 10/101 (9.9%), and 2/47 (4.3%) across the three groups; grade ≥ 2 radiation pneumonitis was lower with Tα1 exposure (18.8% vs. 8.1%, *p* = 0.04).
Zhou 2018 [44]	1. OS; 2. DFS; 3. follow-up and treatment duration; 4. liver function and rejection; 5. AFP and recurrence timing; 6. immune response; 7. tumor recurrence and metastasis	1. OS rates in the treatment group were 100% at 1 year, 94.5% at 3 years, and 77.8% at 5 years vs. 77.8% at 1 year and 0% at 2 years in controls (*p* < 0.001); 2. DFS rates were 88.9% at 1 year, 55.6% at 3 years, and 50.0% at 5 years vs. 22.2% at 1 year in controls (*p* < 0.001); 3. median follow-up was 60 months (47–84) in the treatment group vs. median survival of 15.5 months (9–24) in controls; 4. liver function normalized postoperatively, with no significant difference in rejection rates; 5. Post-LT AFP remained low, and recurrence time was prolonged in the treatment group; 6. FoxP3+ Treg decreased from 1.21% to 0.63% at 1 year, while CD8+ T/CD3+ T indices increased; 7. lung metastasis and relapse were less frequent in the treatment group.	The triple-combination therapy was well tolerated. Rejection rates were 11.1% in the treatment group vs. 22.2% in the control group, with no significant difference (*p* > 0.05), and no severe drug-induced liver or kidney injury was reported during 60-month follow-up.

AE, adverse event; AFP, alpha-fetoprotein; anti-CTLA-4, anti-cytotoxic T-lymphocyte-associated protein 4; CCRT, concurrent chemoradiotherapy; CG, control group; CI, confidence interval; CR, complete response; CRP, C-reactive protein; CTCAE, Common Terminology Criteria for Adverse Events; DCR, disease control rate; DFS, disease-free survival; EGFR-TKI, epidermal growth factor receptor tyrosine kinase inhibitor; GBM, glioblastoma; HR, hazard ratio; IFN-γ, interferon gamma; IL-2, interleukin-2; IL-6, interleukin-6; IPI, ipilimumab; irAE, immune-related adverse event; ITT, intention-to-treat; KPS, Karnofsky Performance Status; LT, liver transplantation; MMSE, Mini-Mental State Examination; MPR, major pathological response; MRI, magnetic resonance imaging; NK, natural killer; NLR, neutrophil-to-lymphocyte ratio; NR, not reported; ORR, objective response rate; OS, overall survival; pCR, pathological complete response; PET, positron emission tomography; PFS, progression-free survival; PR, partial response; PSM, propensity score matching; QOL, quality of life; RCB, residual cancer burden; RECIST, Response Evaluation Criteria in Solid Tumors; RFS, recurrence-free survival; RT, radiotherapy; SAE, serious adverse event; SBRT, stereotactic body radiotherapy; SD, stable disease; Tα1, thymosin alpha 1; TG, treatment group; TNF-α–Tα1, tumor necrosis factor alpha–thymosin alpha 1 fusion protein; Treg, regulatory T cell; ypN0, pathologic node-negative status after neoadjuvant therapy.

## Data Availability

No new data were created or analyzed in this study. Data sharing is not applicable to this article.

## Supplementary Materials

The following supporting information can be downloaded at: https://www.mdpi.com/article/10.3390/ph19091492/s1, Table S1: Search Strategies Applied Across Databases.
