# Supplementary material for: Clinical Applications and Evidence Landscape of Thymosin Alpha 1 as an Immunomodulatory Adjunct in Cancer Care: A Scoping Review
Source: Pharmaceuticals (Basel). 2026 Sep 19;19(9):1492. doi: 10.3390/ph19091492 (PMC13610580; doi:10.3390/ph19091492)
Supplement: Supplementary file 1 [file pharmaceuticals-19-01492-s001.zip › pharmaceuticals-4504307-supplementary.pdf]

SUPPLEMENTARY

Supplementary Table S1. Search Strategies Applied Across Databases

Database	Search Strategy	Rationale for keyword selection	Records Retrieved
**PubMed**	(cancer*[tiab] OR carcinoma*[tiab] OR neoplasm*[tiab] OR tumor*[tiab] OR tumour*[tiab] OR malignanc*[tiab] OR "Neoplasms"[MeSH]) AND ("Thymosin alpha 1"[tiab] OR "Thymosin alpha-1"[tiab] OR "Thymosin alpha1"[tiab] OR "Thymosin a1"[tiab] OR "Thymosin-a1"[tiab] OR "Thymalfasin"[tiab])	Cancer-related keywords cover diagnostic terminology and spelling variants, supplemented by the Neoplasms MeSH term. Intervention terms include thymosin alpha 1, its spelling and hyphenation variants, and the alternative name thymalfasin.	**105**
**EMBASE**	('cancer'/exp OR cancer*:ab,ti OR carcinoma*:ab,ti OR neoplasm*:ab,ti OR tumor*:ab,ti OR tumour*:ab,ti OR malignanc*:ab,ti) AND ('thymosin alpha 1':ab,ti OR 'thymosin alpha-1':ab,ti OR 'thymosin alpha1':ab,ti OR 'thymosin a1':ab,ti OR 'thymosin-a1':ab,ti OR thymalfasin:ab,ti)	The exploded cancer term and cancer-related keywords cover indexed records and title/abstract terminology. Intervention terms include thymosin alpha 1, its spelling and hyphenation variants, and thymalfasin.	**227**
**CENTRAL**	#1 cancer*:ti,ab,kw<br>#2 carcinoma*:ti,ab,kw<br>#3 neoplasm*:ti,ab,kw<br>#4 tumor*:ti,ab,kw<br>#5 tumour*:ti,ab,kw<br>#6 malignanc*:ti,ab,kw<br>#7 #1 OR #2 OR #3 OR #4 OR #5 OR #6<br>#8 "Thymosin alpha 1":ti,ab,kw<br>#9 "Thymosin alpha-1":ti,ab,kw<br>#10 "Thymosin a1":ti,ab,kw<br>#11 "Thymosin-a1":ti,ab,kw<br>#12 #8 OR #9 OR #10 OR #11<br>#13 #7 AND #12	Cancer-related keywords cover diagnostic terminology and spelling variants. Intervention phrases capture alpha/a and hyphenation variants of thymosin alpha 1 in title, abstract, and keyword fields.	**16**
